# A Systematic Review and Meta-Analysis of the Effect of Gamified Clinical Skill Learning on Learning Outcomes in Undergraduate Nursing Education

**DOI:** 10.1097/jnr.0000000000000772

**Published:** 2026-09-25

**Authors:** Wen-Ling CHEN, Siriluk WINITCHAYOTHIN, Chun-Hung CHENG, Santo Imanuel TONAPA, Fan-Hao CHOU, Peta-Anne ZIMMERMAN

**Affiliations:** 1 College of Nursing, Kaohsiung Medical University, Kaohsiung City, Taiwan; 2 Department of Nursing, Kaohsiung Medical University Hospital, Kaohsiung Medical University, Kaohsiung City, Taiwan; 3 Department of Fundamental Nursing, Faculty of Nursing, Mahidol University, Bangkok, Thailand; 4 School of Nursing, Shu-Zen Junior College of Medicine and Management, Kaohsiung City, Taiwan; 5 School of Nursing and Midwifery, Griffith University, Southport, QLD, Australia

**Keywords:** gamification, nursing education, clinical skills, meta-analysis, learning outcomes

## Abstract

**Background:**

Nursing education requires innovative approaches to develop core competencies in knowledge, skills, and attitudes. Gamified learning interventions offer promising alternatives to traditional teaching methods for enhancing outcomes in undergraduate nursing education.

**Purpose:**

This systematic review and meta-analysis was conducted to evaluate the effectiveness of gamified learning interventions on knowledge acquisition, skill performance, and attitude enhancement among undergraduate nursing students and to investigate the differences in effects achieved across different gamification categories.

**Methods:**

Following PRISMA (Preferred Reporting Items for Systematic Reviews and Meta-analyses) guidelines, a comprehensive search was conducted across six databases (A+ Education, CINAHL, Embase, ERIC, PubMed, and Web of Science) on March 18, 2025. This search targeted randomized controlled trials designed to compare gamified learning with traditional teaching methods in undergraduate nursing education. Study quality was assessed using the Cochrane Risk of Bias 2.0 tool, and evidence certainty was evaluated using the GRADE (Grading of Recommendations Assessment, Development, and Evaluation) methodology.

**Results:**

The findings of randomized controlled trials involving a total of 1,698 participants demonstrated significantly positive effects favoring gamified learning across all key domains, including knowledge acquisition (standardized mean difference [SMD]=0.81, 95% CI [0.55, 1.07]), skill performance (SMD=1.64, 95% CI [0.96, 2.32]), and attitude enhancement (SMD=0.54, 95% CI [0.07, 1.00]). The subgroup analysis revealed differential effectiveness across gamification categories, with serious games demonstrating large and consistent effects on knowledge acquisition (SMD=0.84, 95% CI [0.43, 1.25], *I* ^2^=61%), while gamified web and mobile learning interventions showed moderate effects with greater variability (SMD=0.55, 95% CI [0.23, 0.87], *I* ^2^=70%). Skill performance exhibited the largest overall effect sizes, although substantial heterogeneity was observed across all outcome domains (*I* ^2^=82–95%). The results of the GRADE assessment support moderate-certainty evidence for all learning outcomes.

**Conclusions:**

The results of this systematic review and meta-analysis provide robust evidence that gamified learning interventions significantly enhance undergraduate nursing education across the knowledge, skills, and attitudes domains. Serious games demonstrated superior consistency for knowledge acquisition, while the strongest overall effects were identified for skill performance. The substantial heterogeneity observed underscores the critical importance of selecting appropriate gamification approaches based on specific educational objectives, institutional technological capabilities, and implementation contexts. These findings support the strategic integration of evidence-based gamified learning into nursing curricula as an effective supplement to traditional teaching methods. Future research should focus on assessing long-term competency retention and developing standardized implementation frameworks to guide the widespread adoption of effective gamified learning approaches in nursing education.

## Introduction

Core competencies in nursing education encompass the domains of knowledge, attitude, and practice, each of which is essential to delivering high-quality nursing care and aligns with established learning taxonomies (Anderson & Krathwohl, 2001; Lin et al., 2016). A wide range of teaching and learning strategies have been employed to foster these core competencies. Whereas traditional instruction emphasizes face-to-face lectures and demonstrations, more recent pedagogical approaches have incorporated interactive elements such as quizzes, video-based activities, computer games, and online flipped learning models to enhance student engagement and higher-order skills such as critical thinking (Aljezawi & Albashtawy, 2015; Erden & Kaya, 2025; Inangil et al., 2022). These more recent transitional methods have paved the way for the development and implementation of even more structured, theory-driven gamification approaches to learning.

Gamified learning is defined as the use of game elements (e.g., points, levels, badges, leaderboards, and feedback systems) in non-game contexts to increase student motivation, engagement, and learning effectiveness (Böckle et al., 2018; Kinder & Kurz, 2018). Contemporary meta-analytic evidence indicates gamified learning interventions can effectively enhance nursing competency domains, showing significant and positive effects on cognitive learning outcomes, motivational outcomes (including attitude), and behavioral outcomes (including practical skills; Sailer & Homner, 2020).

Nursing education currently employs diverse gamification approaches, including web-based interactive games, mobile applications, virtual reality simulations, serious games, and blended methods that combine traditional teaching with game elements (Chang et al., 2022; Farsi et al., 2021; Koivisto et al., 2018). While evidence in the literature supports that these gamified methods can improve knowledge acquisition, skill performance, and learning attitudes in the context of medical and health care education (Gorbanev et al., 2018; van Gaalen et al., 2021), evidence regarding the comparative effectiveness of these different approaches on the core learning outcomes of undergraduate nursing students remains limited. Also within this field, recent qualitative studies documenting both the perceived enthusiasm and practical challenges faced by nursing educators in using virtual games in nursing curricula stress the importance of educator preparation and implementation support (Ordu & Calişkan, 2024).

Previous systematic reviews have focused primarily on single gamification interventions or examined general effects across various educational domains, with limited evaluation of differential learning outcomes among multiple gamification approaches (Kim & Castelli, 2021; Seo et al., 2024). Moreover, existing research has predominantly focused on individual gamification teaching methods, thus lacking systematic comparative analyses of the relative effectiveness of different formats (Nylén-Eriksen et al., 2025). Given the diversity and complexity of gamification teaching formats, a critical knowledge gap persists regarding how different gamification approaches used in undergraduate nursing education influence the development of students' knowledge, skills, and attitudes.

Therefore, the aims of this systematic review and meta-analysis were, first, to evaluate the effectiveness of various gamified learning interventions on knowledge, skills, and attitudes among undergraduate nursing students and, second, to examine the differential impacts of different gamification formats on these core learning outcomes.

## Methods

### Design

The review protocol of this study was prospectively registered in the Open Science Framework (https://doi.org/10.17605/OSF.IO/SZBFK) and conducted in accordance with the Preferred Reporting Items for Systematic Reviews and Meta-analyses (PRISMA) guidelines (Page et al., 2021).

### Search Strategy

A systematic literature search was conducted with guidance from an experienced health sciences librarian across six electronic databases (A+ Education, CINAHL, Embase, ERIC, PubMed, and Web of Science) from their dates of inception to March 18, 2025. The search strategy employed comprehensive keyword combinations for nursing students (nurs*, student*, undergrad*, bachelor*, pre-license, university*, pre-registrat*), gamification interventions (gami*, game*), learning outcomes (skill*, pract*, performan*, motivat*, attitud*, achiev*), and randomized controlled trial (RCT) methodology (random*, trial*, experimental*). Terms were combined using Boolean operators (AND, OR) with truncation symbols used to maximize retrieval sensitivity.

Database-specific adaptations were implemented to optimize search performance while maintaining conceptual consistency across platforms. The complete search syntax for each database is provided in Tables S1, Supplemental Digital Content 3, https://links.lww.com/JNR/A20 and S2, Supplemental Digital Content 3, https://links.lww.com/JNR/A21. The initial searches applied no language restrictions, with subsequent screening limited to English and Chinese publications. Reference mining of the included studies and relevant systematic reviews were performed to ensure comprehensive identification of the eligible research.

### Eligibility Criteria

The inclusion criteria for articles were determined using the Population, Intervention, Comparison, Outcome, and Study (PICOS) framework (Amir-Behghadami & Janati, 2020). Specifically, studies were included if they were RCTs that recruited participants who were undergraduate nursing students and had a control group undergoing gamified clinical skill learning. Studies were excluded if they were not published in English or Chinese, were not published in a peer-reviewed journal, did not measure learning outcomes, or their results included postgraduate students as participants.

### Study Screening and Selection

All of the retrieved studies were imported into EndNote 20 (Clarivate, Philadelphia, PA, USA), and Covidence (Veritas Health Innovation, Melbourne, Australia) was used to exclude duplicate studies. Two reviewers independently screened the titles and abstracts of the remaining studies for eligibility. The full text of each retained study was screened and assessed for eligibility by the two reviewers on the basis of the aforementioned inclusion and exclusion criteria, with a third reviewer invited to resolve any conflicts of opinion that arose.

### Data Extraction

One reviewer was responsible for extracting the relevant study data (i.e., author name, publication year, country, study design, population/education, interventions, learning modality, type and duration of learning sessions, measurement tools, and outcomes) from the included studies, with the second reviewer consulted when further clarification was necessary.

### Quality Appraisal

The methodological quality of each included RCT was assessed using the Cochrane Risk of Bias 2.0 (RoB 2) tool (Sterne et al., 2019). This tool was designed to evaluate potential bias across the following five domains: bias arising from the randomization process; bias due to deviations from intended interventions; bias due to missing outcome data; bias in measurement of the outcome; and bias in selection of the reported result. Each domain is addressed by a series of signaling questions designed to guide reviewers in making transparent and consistent judgments. Based on the responses to these signaling questions, each domain is rated as having a “low risk of bias,” “some concerns,” or “high risk of bias.” These domain-level judgments are then used to derive an overall risk-of-bias rating for each outcome reported in the trial.

Two reviewers independently applied the RoB 2 tool to each study included in this review, and all judgments and supporting rationales were documented. If discrepancies arose between reviewers, they were first discussed in detail to reach consensus, and when consensus could not be reached, a third reviewer (the review supervisor) was consulted to achieve a resolution. This process ensured transparency, reproducibility, and rigor in assessing the quality of evidence included in this review. The final judgments were used not only to describe the methodological rigor of the included studies but also to inform the Grading of Recommendations Assessment, Development, and Evaluation (GRADE) approach to rating the certainty of the evidence for each outcome. The GRADE framework was used in this study to assess the certainty of the evidence in systematic reviews and health technology assessments and to grade the strength of health recommendations (Santesso et al., 2016).

### Sensitivity Analysis

Sensitivity analyses were conducted in this study to evaluate the robustness of the findings. First, the meta-analyses were repeated after excluding studies judged as at high risk of bias based on the RoB 2.0 assessment. Second, a leave-one-out analysis was conducted, with each included study sequentially omitted to examine its influence on the pooled effect estimates. Finally, the results obtained under the random-effects and fixed-effects models were compared with assess consistency across analytic approaches.

### Data Synthesis and Analysis

The data extracted from each study were transformed into a precalculated effect size using the Campbell Collaboration method (Lipsey & Wilson, 2001), which applies an equation that considers the intervention mean value, standard deviation, and sample size (Elbourne et al., 2002; Lipsey & Wilson, 2001). Given that the reviewed studies used various measurement tools to assess knowledge acquisition, skill performance, and attitudes, the standardized mean difference (SMD) was used to estimate the effect size of each study, interpreted as small (0.2), medium (0.5), or large (0.8; Hedges, 1981).

An SMD with a 95% confidence interval (95% CI) was used to calculate the overall effect size using Cochrane Review Manager Version 5.4.1 software (Cochrane Collaboration, 2020). In addition, heterogeneity was estimated using Cochran’s *Q* (where *p*<.01 was regarded as significant), the tau-squared (τ^2^) statistic, and the *I* ^2^ statistic, the latter of which indicates the percentage of observed variance explained by heterogeneity (Borenstein et al., 2021). Due to the varied contexts of gamification-based learning and the variety of measurement tools employed in the reviewed studies, a random-effects model was applied in this study to avoid underestimating heterogeneity (Borenstein, 2019).

### Handling Multi-Arm Trials

In those included studies such as Farsi et al. (2021) that included multiple arms rather than one intervention group and one control group, each intervention–control comparison was treated as an independent analysis in the meta-analysis. To prevent double-counting of participants in the shared control group, the control sample size was proportionally divided across the comparisons. This analytic strategy adheres to the methodological recommendations outlined in the Cochrane Handbook for Systematic Reviews of Interventions (Higgins et al., 2022) and Campbell Collaboration Practical Meta-Analysis framework (Lipsey & Wilson, 2001) and helps ensure accurate weighting and unbiased effect size estimation.

## Results

### Study Identification and Selection

From the six databases, 1,042 articles were initially identified. After removing 283 duplicates, the titles and abstracts of the remaining 759 were screened, of which 708 were deemed ineligible for failing to meet the PICOS criteria, leaving 51 studies for the full-text review. Subsequently, 32 studies were excluded after the full-text review for the following reasons: 28 did not meet the inclusion criteria, 3 were non-journal articles, and 1 investigated a mixed participant population comprising registered nurses. The final pool available for analysis included 19 articles, all of which met all of the eligibility criteria and provided sufficient data for meta-analysis (Figure S1, Supplemental Digital Content 1, https://links.lww.com/JNR/A18).

### Methodological Quality of the Reviewed Studies

The risk-of-bias assessment using the Cochrane RoB-2 tool revealed that 15 of 19 studies (79%) demonstrated low overall risk, 2 demonstrated some concerns, and 2 were at high risk of bias (Figure S2, Supplemental Digital Content 2, https://links.lww.com/JNR/A19). Aljezawi and Albashtawy (2015) and Al-Mugheed et al. (2022) were identified as high risk due primarily to deviations in implementation from stated intentions. Specifically, Aljezawi and Albashtawy (2015) were judged to be at high risk because participants in the quiz game group did not consistently adhere to the assigned gaming protocol, leading to deviations from the intended intervention. Similarly, Al-Mugheed et al. (2022) was rated at high risk due to variable compliance with the mobile application protocol, with some participants reverting to traditional learning approaches instead of using the gamified platform. These deviations potentially affected intervention delivery fidelity, justifying the high risk of bias judgment in this domain. The results of the domain-specific analysis shown in Figure S2, Supplemental Digital Content 2, https://links.lww.com/JNR/A19 demonstrate low risk for the randomization process in 15 studies, with 4 studies having some concerns regarding sequence generation or allocation concealment. Notably, missing outcome data demonstrated consistently low risk across all of the included studies. Measurement of outcomes showed low risk in 13 studies, with 6 studies raising concerns about assessor blinding. The selection of reported results revealed a low risk in 17 studies. Despite these methodological limitations, the overall evidence quality was deemed sufficient for reliable meta-analytic synthesis.

The GRADE evidence assessment of the 19 RCTs revealed moderate certainty with consistent positive effects favoring gamified learning across all outcomes (Table S3, Supplemental Digital Content 3, https://links.lww.com/JNR/A22). Knowledge outcomes (17 RCTs, *n*=1,505) demonstrated large effects (SMD=0.81, 95% CI [0.55, 1.07]), while skill outcomes (10 RCTs, *n*=856) showed the strongest impact (SMD=1.64, 95% CI [0.96, 2.32]). Attitude outcomes (4 RCTs, *n*=434) exhibited moderate positive effects (SMD=0.54, 95% CI [0.07, 1.00]). All of the domains received moderate certainty ratings due to substantial heterogeneity (*I* ^2^=82–95%), reflecting variations in intervention designs, assessment methodologies, and implementation contexts that could not be adequately explained through subgroup analyses. These findings confirm the superiority of gamified learning over traditional approaches in undergraduate nursing education, although cautious interpretation of the results is warranted in light of the between-study variability detailed in Table S3, Supplemental Digital Content 3, https://links.lww.com/JNR/A22.

### Study Characteristics

A summary of the characteristics of the 19 included RCTs is presented in Table S4, Supplemental Digital Content 3, https://links.lww.com/JNR/A23. All of the included studies recruited undergraduate nursing students and were published between 2012 and 2025. The studies were conducted across multiple countries, with the largest representation from Turkey (seven studies: Aktaş et al., 2024; Bayram & Caliskan, 2019; DemİRay & AÇIl, 2024; Inangil et al., 2022; Kulakaç & Çilingir, 2024; Ordu & Çalışkan, 2023; Özkan Şat et al., 2025), followed by China and Iran (two studies each: Ma et al., 2021; Zhao et al., 2025, and Farsi et al., 2021; Nasirzade et al., 2024), and individual studies from eight other countries: Jordan (Aljezawi & Albashtawy, 2015), Cyprus (Al-Mugheed et al., 2022); Spain (Gutierrez-Puertas et al., 2021); France (Blanié et al., 2020); Taiwan (Chang et al., 2022); Canada (Verkuyl et al., 2017); the United States (LeFlore et al., 2012); Egypt (Elzeky et al., 2022). The total sample comprised 1,698 participants (intervention group=849; control group=849). Substantial heterogeneity was observed across these studies in terms of gamification content design, implementation duration, and learning outcome measurement tools.

### Effects of Gamified Clinical Skill Learning

#### Knowledge outcomes

**Figure 1:**
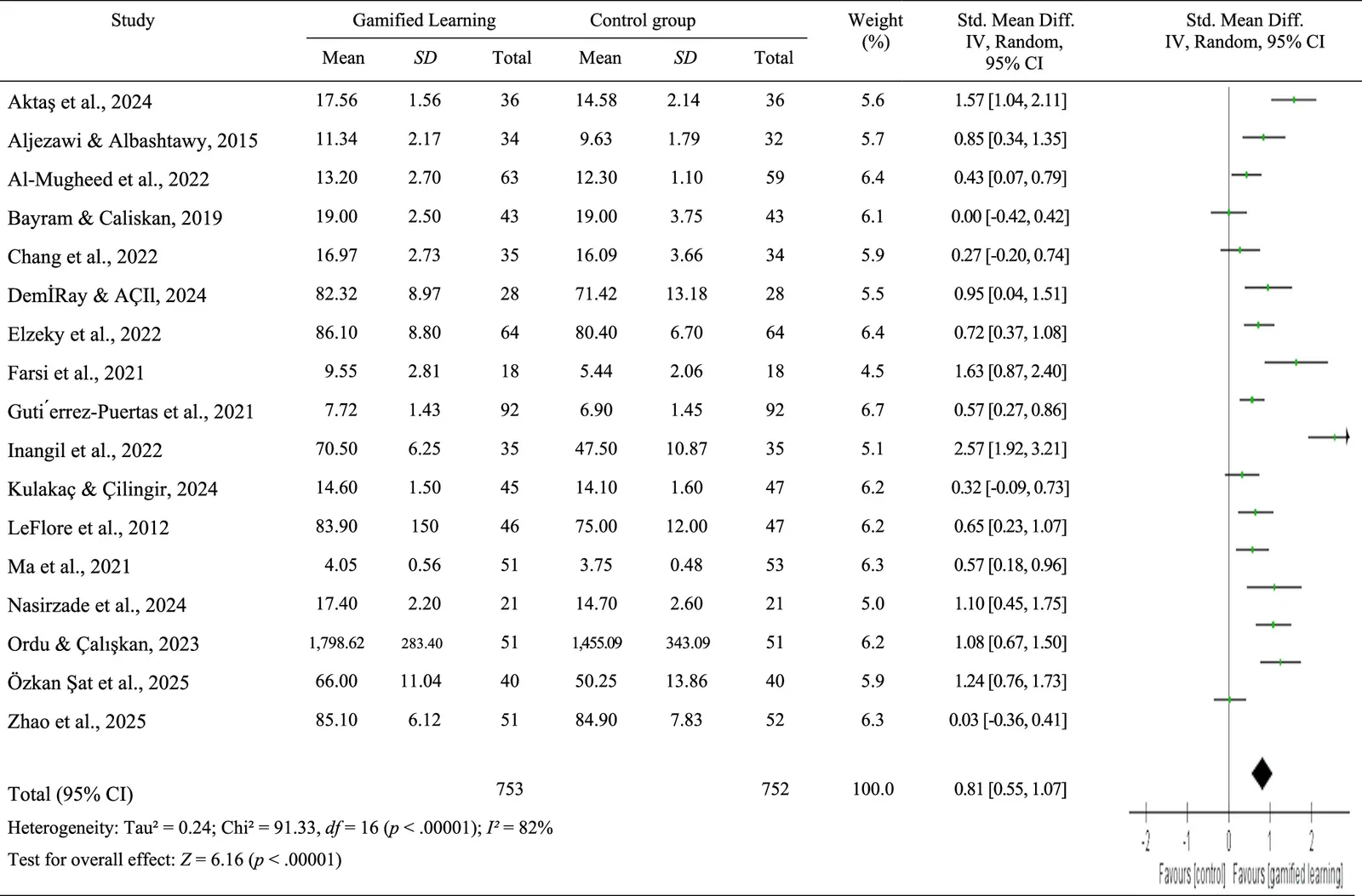
Meta-Analysis of the Effects of Gamified Clinical Skill Learning on Knowledge Acquisition *Note.* Std. mean diff. = standard mean difference.

A meta-analysis of 17 studies comprising 1,505 students was conducted to examine the effects of gamified clinical skill learning on knowledge acquisition. The random-effects model revealed a pooled standardized mean difference of 0.81 (95% CI [0.55, 1.07], *p*<.001), indicating a large positive effect favoring gamified interventions. However, substantial heterogeneity was observed across these studies (*τ*²=0.24, χ^2^=91.33, *df*=16, *p*<.001, *I* ^2^=82%; Figure 1), suggesting considerable variation in intervention effects and study characteristics.

#### Skill performance outcomes

**Figure 2:**
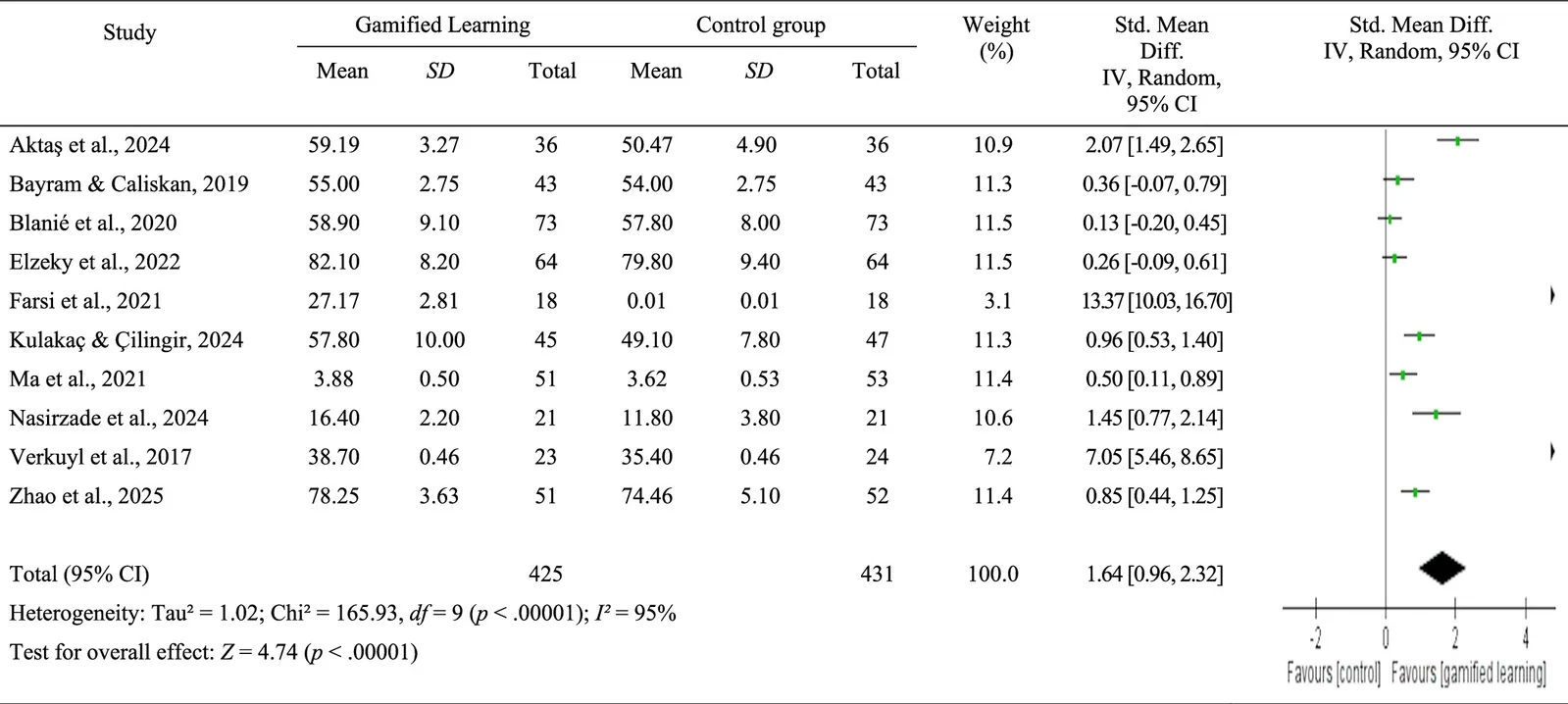
Meta-Analysis of the Effects of Gamified Clinical Skill Learning on Skill Performance *Note.* Std. mean diff. = standard mean difference.

A meta-analysis of 10 studies comprising 856 students was used to assess the impact of gamified learning on skill performance. The pooled analysis demonstrated a large effect size with a standardized mean difference of 1.64 (95% CI [0.96, 2.32], *p*<.001). The presence of substantial heterogeneity (*τ*²=1.02, χ²=165.93, *df*=9, *p*<.001, *I*²=95%; Figure 2) is indicative of considerable variability in the effect of the gamified interventions on skills development across study contexts and methodologies.

#### Attitude outcomes

**Figure 3:**
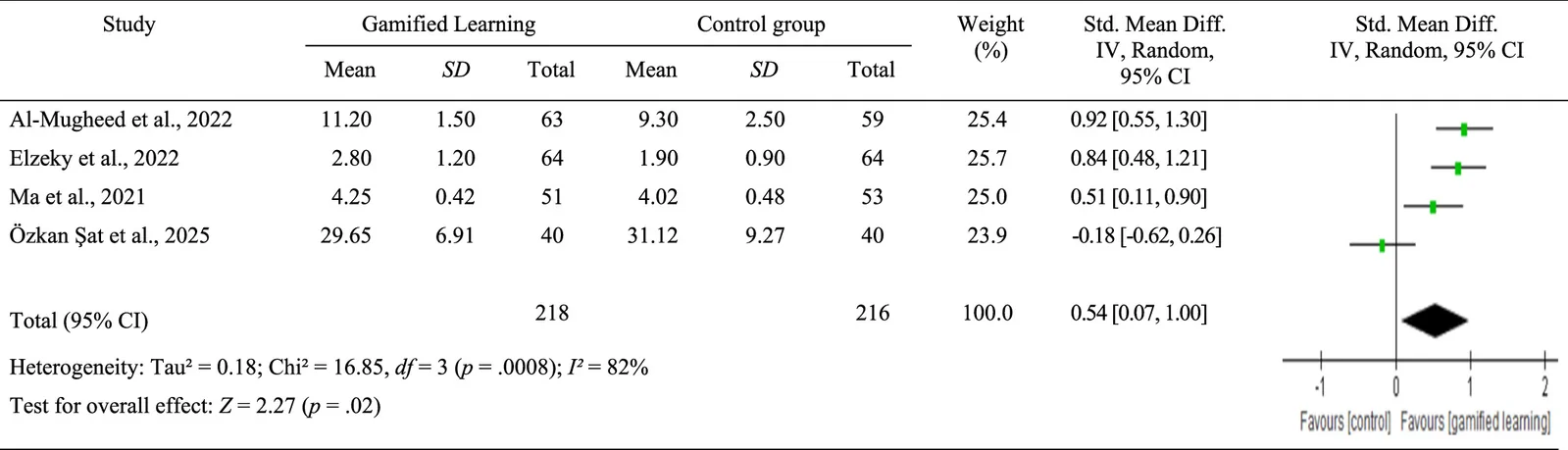
Meta-Analysis of the Effects of Gamified Clinical Skill Learning on Attitude *Note.* Std. mean diff. = standard mean difference.

A meta-analysis of four studies comprising 434 students was used to assess attitude changes following gamified clinical skill learning interventions. The results, showing a moderate pooled effect size of 0.54 (95% CI [0.07, 1.00], *p*=.02) with substantial heterogeneity (*τ*²=0.18, χ^2^=16.85, *df*=3, *p*<.001, *I*²=82%; Figure 3), demonstrated positive effects across these studies. This finding suggests gamified learning approaches enhance student attitudes toward clinical skill learning significantly more than traditional educational methods, although notable variation in effect magnitude was observed among the included studies.

These findings confirm that gamified clinical skill-learning interventions effectively enhance undergraduate nursing education across the knowledge acquisition, skill performance, and attitude domains. Skill performance demonstrated the largest effect sizes, while knowledge and attitude outcomes showed significantly positive improvements. The substantial heterogeneity across all domains indicates that intervention effectiveness depends on design characteristics and implementation contexts, necessitating the careful selection of gamified learning strategies based on specific educational objectives and institutional capabilities.

### Subgroup Analysis by Gamification Categories and Knowledge Outcomes

**Figure 4:**
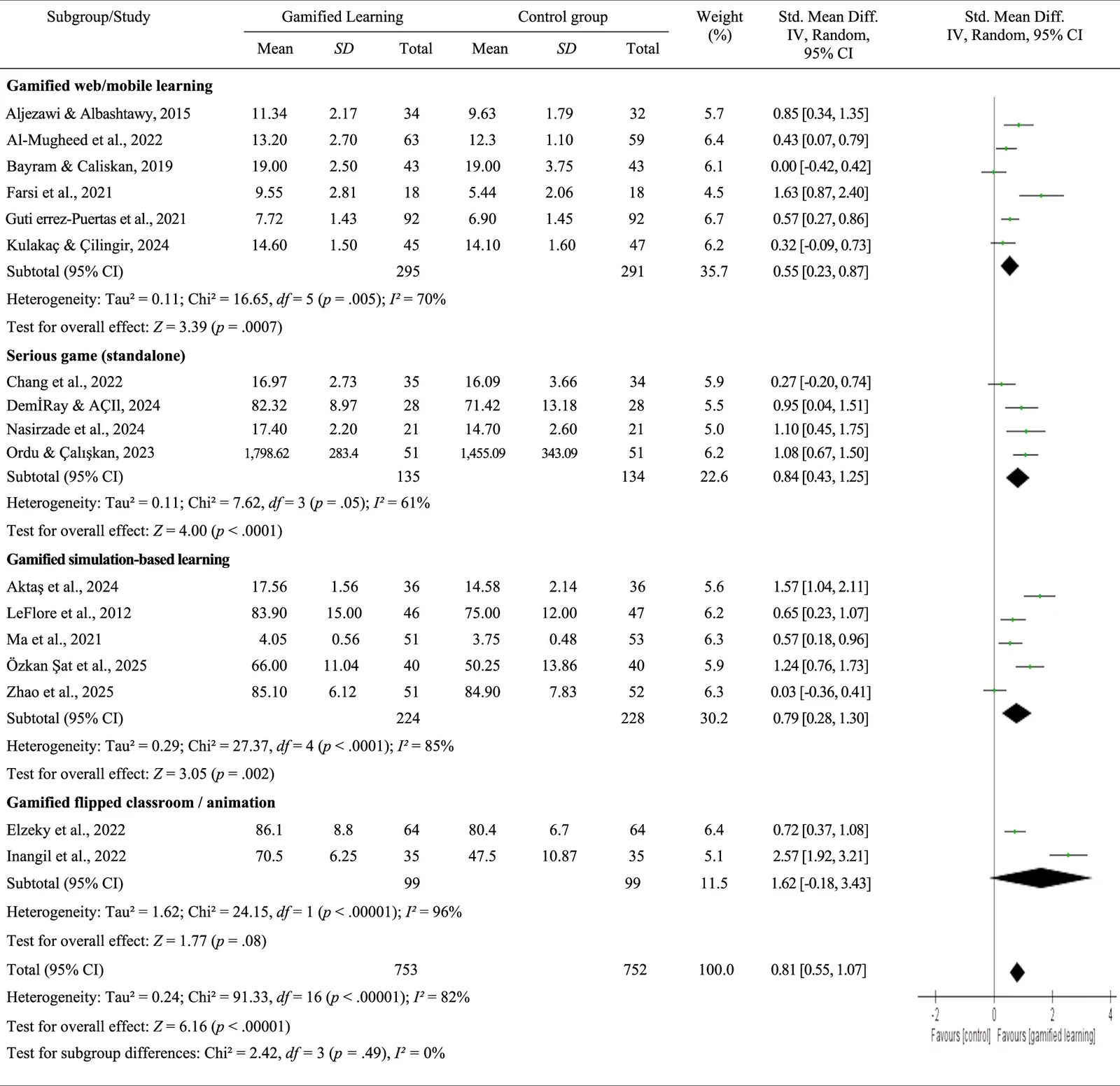
Subgroup Meta-Analysis of Associations of Gamification Categories With Knowledge *Note.* Std. mean diff. = standard mean difference.

The meta-analysis of 17 studies involving 1,505 students conducted for this study revealed differential effects on knowledge acquisition across four gamification categories (Figure 4). All of the intervention types demonstrated statistically significant, positive effects relative to control conditions, although effect magnitudes and between-study consistency varied substantially across categories.

#### Gamified web/mobile learning

These interventions (six studies, *n*=295) exhibited a moderate pooled effect (SMD=0.55, 95% CI [0.23, 0.87]) with substantial heterogeneity (*I* ^2^=70%). They incorporated web-based or mobile platforms with embedded gamification elements, including quizzes, badges, and leaderboards. The moderate effects with considerable variability achieved suggest that, while scalable digital tools provide meaningful learning enhancement, outcomes depend significantly on specific design features and implementation quality.

#### Serious games (standalone)

These interventions (four studies, *n*=135) exhibited a large effect size (SMD=0.84, 95% CI [0.43, 1.25]) with moderate heterogeneity (*I* ^2^=61%). The purpose-built educational games used in these studies integrated learning content within immersive narrative and simulation environments. The substantial effects achieved indicate that standalone serious games consistently induce meaningful cognitive engagement, although some variation was detected across different game designs and educational contexts.

#### Gamified simulation-based learning

These interventions (five studies, *n*=224) exhibited a large effect (SMD=0.79, 95% CI [0.28, 1.30]) with substantial heterogeneity (*I* ^2^=85%). The considerable variability suggests significant differences in intervention design quality or implementation approaches, with gamification additions potentially interacting differently with existing simulation-based learning frameworks across various educational settings.

#### Gamified flipped classroom/animation

These interventions (two studies, *n*=99) exhibited a large effect size (SMD=1.62, 95% CI [0.18, 3.43]) with extremely high heterogeneity (*I* ^2^=96%). However, the confidence interval crossing zero indicates nonsignificant effects, and the substantial heterogeneity reflects considerable differences between the limited number of studies in this category, necessitating cautious interpretation of these preliminary findings.

The subgroup analysis reveals that serious games and gamified simulation-based learning achieved the most robust knowledge acquisition effects, with large effect sizes. Gamified web/mobile learning demonstrated consistent, moderate benefits, while gamified flipped classroom approaches showed inconclusive results due to the limited number of studies and high variability. These findings indicate that intervention selection should prioritize evidence-based approaches with demonstrated effectiveness patterns. However, formal statistical testing of between-subgroup differences was not conducted, necessitating cautious interpretation of the findings and indicating the need for future meta-regression analyses to examine moderating factors systematically.

### Sensitivity Analyses

The results of the sensitivity analyses confirm the stability of the findings in this study. When excluding the two studies identified with a high risk of bias (Aljezawi & Albashtawy, 2015; Al-Mugheed et al., 2022), the pooled effect sizes for knowledge, skills, and attitudes remained materially unchanged, further supporting the robustness of the results. Furthermore, the leave-one-out analysis demonstrated that no single study excessively influenced the overall pooled outcomes or primarily accounted for the observed heterogeneity (knowledge *I* ^2^=82%, skills *I* ^2^=95%). Moreover, the fixed-effect model yielded effect directions consistent with those under the random-effects model, although the effect size estimates were slightly attenuated. Collectively, these findings suggest the stability of the conclusions across different analytic assumptions.

## Discussion

This review was implemented to determine whether gamified learning improves knowledge, skills, and attitudes in undergraduate nursing education, and whether the related effects differ by gamification format. The pooled results indicate meaningful benefits across all three domains, with large improvements in knowledge (SMD=0.81), particularly strong gains in skills (SMD=1.64), and moderate gains in attitudes (SMD=0.54). These findings place gamification as a pedagogical approach that complements, rather than merely decorating, traditional methods in nursing education, which is a conclusion consistent with broader syntheses showing the positive cognitive, motivational, and behavioral effects of gamified learning in medical and health care education (Sailer & Homner, 2020; van Gaalen et al., 2021).

Knowledge gains at the magnitude identified in this review indicate structured game elements can reliably raise test performance over the level achievable through standard classroom instruction methods, despite the observed presence of heterogeneity. Serious games are a plausible driver of these consistent cognitive benefits because they embed content within purposeful scenarios, clear feedback loops, and a mastery-oriented progression, which are features that have been regularly linked in nurse education reviews and trials to better knowledge acquisition (Min et al., 2022; Thangavelu et al., 2022). The pattern aligns with design frameworks that emphasize alignment of points, levels, and feedback with learning objectives rather than superficial reward mechanics (Böckle et al., 2018; Kinder & Kurz, 2018).

Skill outcomes showed the largest effects in the meta-analysis, which fits theory and practice. Purposefully designed games and simulated environments afford repeated practice, immediate feedback, and safe error tolerance, all of which are ingredients known to accelerate procedural learning and transfer in clinical education (Böckle et al., 2018; Min et al., 2022; Thangavelu et al., 2022). The differences in skill effects observed across interventions, reflected in substantial heterogeneity, point to variability in fidelity, alignment with course outcomes, and integration of feedback rules. Notably, the effects appear strongest in those interventions in which progression, feedback specificity, and scenario difficulty are tightly coupled to competency milestones, a pattern echoed in prior evaluations of serious and simulation-based games for nursing competencies (Min et al., 2022; Thangavelu et al., 2022).

Attitude improvements were found to be moderate and positive, although these estimates were grounded on fewer studies than available for the other domains. The observed motivational gains are consistent with prior work showing that leaderboards, badges, and timely feedback can enhance engagement and confidence when they are transparently tied to mastery rather than extrinsic scoring alone (Kinder & Kurz, 2018; Sailer & Homner, 2020). Given the smaller evidence base available, conducting more trials that use standardized attitude measures and report implementation details may be expected to sharpen precision.

The observed subgroup signals for knowledge outcomes provide pragmatic guidance. The standalone serious games were found to yield large effects with moderate heterogeneity, suggesting relatively reliable cognitive benefits when games are purpose-built around realistic clinical narratives and feedback systems (Min et al., 2022; Thangavelu et al., 2022). Also, the gamified simulations showed large effects, albeit with substantial variability, which is consistent with the idea that adding points and rewards to existing simulations helps only when rules, feedback timing, and mastery thresholds are designed coherently (Böckle et al., 2018). The web and mobile formats produced moderate average gains with substantial heterogeneity, a pattern that likely reflects differences in how feedback, progression, and social comparison are implemented at scale (Kinder & Kurz, 2018). Finally, as flipped-classroom and animation-based gamification showed imprecise estimates across a few trials only, inferences based on these results remain tentative.

Certainty and robustness align with these patterns. Most of the included RCTs were associated with a low risk of bias. Although two were classified at high risk due to deviations from intended interventions, excluding them from the analyses did not materially change the pooled effects. Also, the results of the leave-one-out analyses showed no single study dominated the results and that fixed-effect models preserved effect directions. The downgrade to moderate certainty across outcomes was driven chiefly by heterogeneity rather than study flaws, supporting cautious confidence in the positive direction of effects while acknowledging variability across designs and contexts (Santesso et al., 2016; Sterne et al., 2019).

Implementation should flow from these signals and from institutional readiness. Given their consistent effects and structured design, serious games are a strong candidate for reliable knowledge gains where licensing or development support exists (Min et al., 2022; Thangavelu et al., 2022). For rapid improvement in procedural competencies, simulation enhanced with coherent gamification can be powerful. However, teams should pilot, monitor fidelity, and iterate on reward and feedback structures that map directly to competency checklists (Böckle et al., 2018). In resource-constrained settings, scalable web and mobile approaches remain attractive, provided the design includes clear mastery goals, timely feedback, and assessment alignment (Kinder & Kurz, 2018). Across formats, successful adoption was found to depend on technology infrastructure, technical support, and educator preparation, which mirrors prior implementation reviews of game-based learning in nursing (Kuruca Ozdemir & Dinc, 2022) and aligns with qualitative findings that nurse educators value virtual games but require planning, professional support, and dedicated training to use them effectively (Ordu & Calişkan, 2024).

The evidence base still reflects important gaps. Heterogeneity was substantial in every domain, follow-up periods tended to be short, and attitude outcomes were measured in only a few of the studies. These limitations explain the moderate certainty ratings and point to concrete directions for future research. Follow-on research in this field should include head-to-head comparisons of design elements such as feedback schedules and progression rules, the use of common outcome instruments to reduce measurement variance, the routine reporting of implementation fidelity, and longer follow-ups to test the retention and resilience of competencies beyond the immediate posttest period.

In summary, gamified learning significantly improves knowledge, skills, and attitudes in undergraduate nursing education, with the largest and most policy-relevant gains in the skill performance domain, and the most consistent knowledge benefits accrued from serious games. The observed variance in effects based on design and context indicates that educators should match formats to objectives, invest in faculty development, and pilot before scaling. With these steps, gamification may be integrated as an effective complement to conventional teaching and better prepare students for technology-rich clinical environments.

### Conclusions

The findings of this systematic review and meta-analysis demonstrate a significant enhancement effect of gamified learning interventions on undergraduate nursing education across the domains of knowledge acquisition, skill performance, and attitude. Skill performance exhibited the largest effects, while serious games demonstrated the most consistent outcomes across diverse implementation contexts.

The substantial heterogeneity observed across all learning domains indicates intervention effectiveness depends critically on design characteristics, implementation quality, and degree of alignment with specific educational objectives. This variability underscores the importance of referencing the current evidence when selecting gamification approaches to optimally match institutional technological capabilities with the level of educator preparation.

The findings support the strategic integration of gamified learning into nursing curricula as an effective supplement to traditional teaching methods. Implementation success requires systematic consideration of institutional infrastructure, comprehensive educator training, and rigorous evaluation frameworks. Educational leaders should prioritize evidence-based approaches while maintaining the flexibility necessary to adapt interventions based on specific educational contexts and available resources.

As nursing education evolves to meet contemporary health care demands, gamified learning offers valuable tools for enhancing student engagement and preparing them to adapt quickly to complex clinical environments. Future investigations should focus on assessing long-term competency retention, identifying the optimal design characteristics of learning interventions, and developing standardized implementation protocols to guide the widespread adoption of effective gamified learning approaches in nursing education.

### Limitations

This systematic review and meta-analysis has several limitations that warrant careful consideration when interpreting the findings. First, the substantial heterogeneity observed across all of the outcome domains (*I* ^2^=82–95%) represents a fundamental limitation that could not be adequately explained in the subgroup analyses. This heterogeneity, which reflects the significant variation in intervention designs, implementation contexts, assessment methodologies, and study populations, may limit the generalizability of the pooled effect estimates.

Second, the included studies reflected considerable methodological diversity in their gamification approaches, including intervention duration (ranging from single sessions to multi-week implementations), the technological platform used, and the educational context. This methodological heterogeneity may obscure the identification of optimal design characteristics and implementation strategies, thereby limiting the specificity of evidence-based recommendations in educational practice.

Third, most of the included studies employed relatively small sample sizes and short-term follow-up periods. Thus, long-term knowledge retention, skill maintenance, and sustained attitude changes were inadequately assessed. The absence of extended follow-up data limits scholarly understanding of the durability of gamified learning and its capacity to produce lasting educational benefits beyond the immediate post-intervention assessments.

Fourth, the scope of this review was limited to studies published in English or Chinese, potentially introducing language bias and excluding relevant research in other languages. In addition, the focus on RCTs, while methodologically rigorous, may have excluded valuable insights into the effectiveness of gamification reported in quasi-experimental and mixed-methods studies.

Fifth, the risk-of-bias assessment in this study revealed methodological concerns in several studies, particularly regarding the blinding of outcome assessors and potential deviations from intended interventions. These limitations on study quality may affect the reliability of individual study findings and consequently influence the overall meta-analytic conclusions.

Finally, the limited number of studies reporting attitude outcomes (four studies) and the preliminary nature of findings for certain gamification categories, particularly gamified flipped classroom approaches (two studies), restrict the strength of any conclusions drawn for these specific domains and intervention types.

## Supplementary Material
