# Supplementary material for: A Systematic Review and Meta-Analysis of the Effect of Gamified Clinical Skill Learning on Learning Outcomes in Undergraduate Nursing Education
Source: J Nurs Res. 2026 Sep 25;34(5):e484. doi: 10.1097/jnr.0000000000000772 (PMC13608266; doi:10.1097/jnr.0000000000000772)
Supplement: Supplementary file 1 [file jnr-34-e484-s006.pdf]

Figure S2

Cochrane Risk-of-Bias Tool (Version 2) Study Quality Assessment Results

Study	Risk of Bias Domains					
	D1	D2	D3	D4	D5	Overall
Aljezawi & Albashtawy (2015)	-	X	+	-	-	X
Al-Mugheed et al. (2022)	-	X	+	-	-	X
Aktaş et al. (2024)	-	+	+	-	+	-
Bayram & Caliskan (2019)	+	-	+	-	+	-
Blanié et al. (2020)	+	+	+	+	+	+
Chang et al. (2022)	+	+	+	-	+	+
DemİRay & AÇIl (2024)	+	+	+	-	+	+
Elzeky et al. (2022)	+	+	+	-	+	+
Farsi et al. (2021)	+	+	+	+	+	+
Guti´errez-Puertas et al. (2021)	+	+	+	+	+	+
Inangil et al. (2022)	+	+	+	+	+	+
Kulakaç & Çilingir (2024)	+	+	+	-	+	+
LeFlore et al. (2012)	+	+	+	+	+	+
Ma et al. (2021)	-	+	+	-	-	-
Nasirzade et al. (2024)	+	+	+	+	+	+
Ordu & Çalışkan (2023)	+	+	+	-	+	+
Özkan Şat et al. (2025)	+	+	+	+	+	+
Verkuyl et al. (2017)	+	+	+	+	+	+
Zhao et al. (2025)	+	+	+	-	+	+

Note. Domains: D1 = bias arising from the randomization process; D2 = bias due to deviations from intended intervention; D3 = bias due to missing outcome data; D4 = bias in measurement of the outcome; D5 = bias in selection of the reported result.

Judgement

- X High
- \- Some concerns
- \+ Low
