# Supplementary material for: A Systematic Review and Meta-Analysis of the Effect of Gamified Clinical Skill Learning on Learning Outcomes in Undergraduate Nursing Education
Source: J Nurs Res. 2026 Sep 25;34(5):e484. doi: 10.1097/jnr.0000000000000772 (PMC13608266; doi:10.1097/jnr.0000000000000772)
Supplement: Supplementary file 2 [file jnr-34-e484-s001.docx]

**Figure S1**

*PRISMA (Preferred Reporting Items for Systematic Reviews and Meta-Analyses)-Based Flow Diagram of the Review Process*

**Identification of studies via databases and registers**

Records identified from*:

Databases (*N* = 1,042)

A+ Education (*n* = 2)

CINAHL (*n* = 103)

Embase (*n* = 456)

ERIC (*n* = 19)

PubMed (*n* = 46)

Web of Science (*n* = 416)

Records removed before screening:

Duplicate records removed (*n* = 283)

**Identification**

Irrelevant studies (*n* = 708)

Records screened (*n* = 759)

Reports sought for retrieval

(*n* = 51)

Reports not retrieved (*n* = 0)

**Screening**

Reports excluded (*n* = 32):

Wrong outcomes (*n* = 12)

Wrong population (*n* = 1)

Wrong intervention (*n* = 1)

Wrong study design (*n* = 15)

Non-journal articles (*n* = 3)

Reports assessed for eligibility

(*n* = 51)

Total studies included in review (*n* = 19)

**Included**

Studies included in meta-analysis (*n* = 19)
