# Supplementary material for: A Systematic Review and Meta-Analysis of the Effect of Gamified Clinical Skill Learning on Learning Outcomes in Undergraduate Nursing Education
Source: J Nurs Res. 2026 Sep 25;34(5):e484. doi: 10.1097/jnr.0000000000000772 (PMC13608266; doi:10.1097/jnr.0000000000000772)
Supplement: Supplementary file 3 [file jnr-34-e484-s002.docx]

**Table S2**

*Search Strategy*

| Source | Search Strategy | No. of Hits Retrieved |
| --- | --- | --- |
| A+ Education | (Nurs*) AND (student* OR undergrad* OR bachelor* OR pre-license OR “pre license” OR university* OR pre-registrat* OR “pre registra*”) AND (Gami* OR game*) AND (Skill* OR pract* OR performan* OR Motivat* OR Attitud* OR achiev*) AND (RCT OR random* OR trial* OR experimental*) | 2 |
| CINAHL | (Nurs*) AND (student* OR undergrad* OR bachelor* OR pre-license OR “pre license” OR university* OR pre-registrat* OR “pre registra*”) AND (Gami* OR game*) AND (Skill* OR pract* OR performan* OR Motivat* OR Attitud* OR achiev*) AND (RCT OR random* OR trial* OR experimental*) | 104 |
| Embase | (Nurs*) AND (student* OR undergrad* OR bachelor* OR pre-license OR “pre license” OR university* OR pre-registrat* OR “pre registra*”) AND (Gami* OR game*) AND (Skill* OR pract* OR performan* OR Motivat* OR Attitud* OR achiev*) AND (RCT OR random* OR trial* OR experimental*) | 456 |
| ERIC | (Nurs*) AND (student* OR undergrad* OR bachelor* OR pre-license OR “pre license” OR university* OR pre-registrat* OR “pre registra*”) AND (Gami* OR game*) AND (Skill* OR pract* OR performan* OR Motivat* OR Attitud* OR achiev*) AND (RCT OR random* OR trial* OR experimental*) | 19 |
| PubMed | ("nurs*"[All Fields] AND ("student*"[All Fields] OR "undergrad*"[All Fields] OR "bachelor*"[All Fields] OR "pre-license"[All Fields] OR "pre-license"[All Fields] OR "university*"[All Fields] OR "pre registrat*"[All Fields] OR "pre registra*"[All Fields]) AND ("gami*"[All Fields] OR "game*"[All Fields]) AND ("skill*"[All Fields] OR "pract*"[All Fields] OR "performan*"[All Fields] OR "motivat*"[All Fields] OR "attitud*"[All Fields] OR "achiev*"[All Fields]) AND ("RCT"[All Fields] OR "random*"[All Fields] OR "trial*"[All Fields] OR "experimental*"[All Fields])) AND ((meta-analysis[Filter] OR systematicreview[Filter]) AND (fft[Filter])) | 46 |
| Web of Science | ((((ALL=(Nurs*)) AND ALL= (student* OR undergrad* OR bachelor* OR pre-license OR “pre license” OR university* OR pre-registrat* OR “pre registra*”)) AND ALL= (Gami* OR game*)) AND ALL=(Skill* OR pract* OR performan* OR Motivat* OR Attitud* OR achiev*)) AND ALL= (RCT OR random* OR trial* OR experimental*) | 416 |

*Note.* Final Screening: March 18, 2025.
