# Supplementary material for: A Systematic Review and Meta-Analysis of the Effect of Gamified Clinical Skill Learning on Learning Outcomes in Undergraduate Nursing Education
Source: J Nurs Res. 2026 Sep 25;34(5):e484. doi: 10.1097/jnr.0000000000000772 (PMC13608266; doi:10.1097/jnr.0000000000000772)
Supplement: Supplementary file 4 [file jnr-34-e484-s003.docx]

**Table S3**

*GRADE Evidence Profile: Effects of Gamified Learning Interventions on Knowledge, Skills, and Attitudes in Undergraduate Nursing Education*

| Outcome | No. of  Participants (studies) | Follow Up | Certainty of the Evidence (GRADE) | Relative Effect /95% CI | Absolute Effect | Comments |
| --- | --- | --- | --- | --- | --- | --- |
| Knowledge | 1505  (17 RCTs) | Immediate post-test | ⨁⨁⨁◯  Moderate | SMD 0.81/  [0.55, 1.07] | Favours gamified learning | High heterogeneity (*I*² = 82%); downgraded due to inconsistency |
| Skill | 856  (10 RCTs) | Immediate post-test | ⨁⨁⨁◯  Moderate | SMD 1.64/  [0.96, 2.32] | Favours gamified learning | High heterogeneity (*I*² = 95%); downgraded due to inconsistency |
| Attitude | 434  (4 RCTs) | Immediate post-test | ⨁⨁⨁◯  Moderate | SMD 0.54/  [0.07, 1.00] | Favours gamified learning | High heterogeneity (*I*² = 82%); downgraded due to inconsistency |

*Note.* GRADE = Grading of Recommendations Assessment, Development, and Evaluation; SMD *=* standardized mean difference; RCT = randomized controlled trial. Effect sizes interpreted according to Cohen's conventions: 0.2 = small effect, 0.5 = moderate effect, 0.8 = large effect. Positive values favor gamified learning interventions over traditional learning approaches.

**Certainty assessment:** Evidence downgraded one level for serious inconsistency when heterogeneity (*I*² > 50%) indicated substantial between-study variance that could not be adequately explained through subgroup analyses or sensitivity analyses.

**Risk of bias summary**: Overall methodological quality assessed using Cochrane Risk of Bias 2.0 tool. Majority of included studies demonstrated low to moderate risk of bias across five domains. No outcome was downgraded for serious risk of bias concerns.

**GRADE Working Group grades of evidence**: High certainty indicates very high confidence that the true effect lies close to the estimate. Moderate certainty indicates moderate confidence in the effect estimate, with the true effect likely close to the estimate but possibly substantially different. Low certainty indicates limited confidence in the effect estimate, with the true effect potentially substantially different from the reported estimate. Very low certainty indicates very little confidence in the effect estimate, with the true effect likely to be substantially different from the reported estimate.
