# Supplementary material for: A Systematic Review and Meta-Analysis of the Effect of Gamified Clinical Skill Learning on Learning Outcomes in Undergraduate Nursing Education
Source: J Nurs Res. 2026 Sep 25;34(5):e484. doi: 10.1097/jnr.0000000000000772 (PMC13608266; doi:10.1097/jnr.0000000000000772)
Supplement: Supplementary file 5 [file jnr-34-e484-s004.docx]

**Table S1**

*PICO (Population, Intervention, Comparison, Outcome, and Study) Worksheet*

| Item | Object | Keywords |
| --- | --- | --- |
| Population | Nursing student | Nurs*  student*; undergrad*; bachelor*; pre-license; pre license; university*; pre-registrat*;  pre registra* |
| Intervention | Gamification | Gami*; game* |
| Comparison | Usual learning method | **－** |
| Outcomes | Learning outcome | Skill*; pract*; performan*; Motivat*; Attitud*; achiev* |
| Study design | Experimental study | RCT; random*; trial*; experimental* |

*Note.* RCT = randomized controlled trial.
