# Supplementary material for: A Systematic Review and Meta-Analysis of the Effect of Gamified Clinical Skill Learning on Learning Outcomes in Undergraduate Nursing Education
Source: J Nurs Res. 2026 Sep 25;34(5):e484. doi: 10.1097/jnr.0000000000000772 (PMC13608266; doi:10.1097/jnr.0000000000000772)
Supplement: Supplementary file 6 [file jnr-34-e484-s005.docx]

**Table S4**

*Characteristics of the Included Studies: Randomized Controlled Trials of Gamified Learning Interventions in Undergraduate Nursing Education*

|  | Gamification  Category | Author/  Country | Study Design /Sample Size | Participant  Characteristic | Intervention | Gamification Modality/Session and Duration of Intervention | Outcome  Measurement | Main Finding |
| --- | --- | --- | --- | --- | --- | --- | --- | --- |
|  | Gamified web/  mobile learning | 1. Aljezawi &  Albashtawy (2015)/  Jordan | RCT / 66  (EG = 34;  CG = 32) | Fourth-year  nursing  students | Participants played a quiz game (Jeopardy!-style game) that involved organizing patient care. | Quiz game/ 4 months (one semester) | Level of knowledge: 15 topic-specific multiple-choice questions. | Students in the quiz game group achieved significantly higher knowledge scores. |
|  | Gamified web/  mobile learning | 2. Al-Mugheed  et al. (2022)/  Cyprus | RCT / 122  (EG = 63; CG = 59) | Third- and fourth-year nursing  students | Participants received online education and used a game-based mobile application aimed at enhancing their knowledge, attitudes, and compliance with standard precautions. | Game-based mobile application/ 5 weeks | Knowledge of standard precautions is assessed using 18 items, and attitudes are assessed using 16 items.  The checklist for compliance with standard precautions comprises 50 items. | The online education and game-based mobile application improved the participants’ knowledge and attitudes with respect to standard precautions.  The game-based virtual reality phone application group obtained higher mean scores for the compliance domains relative to the traditional lecture group. |
|  | Gamified web/  mobile learning | 3. Bayram & Caliskan (2019)/  Turkey | RCT / 86  (EG = 43;  CG = 43) | First-year  nursing students | Participants used a game-based virtual reality mobile application focused on tracheostomy care. The application involved six stages: preparing materials and suctioning, preparing materials for the inner cannula, preparing materials for peristomal skincare, suctioning, cleaning the inner cannula, and conducting peristomal skincare. | Virtual reality mobile application / 7 days | Tracheostomy Care Knowledge: 23 five-option multiple-choice questions.  Tracheostomy Care Skills Checklists: An objective structured clinical examination.  Objective structure clinical examination time. | No significant difference was observed between preintervention knowledge scores and postintervention knowledge scores even though the postintervention knowledge scores of the experimental groups were higher.  The postintervention skill performance scores of the students in the experimental group were significantly higher than those of the control group. |
|  | Gamified web/  mobile learning | 4. Farsi et al. (2021)/  Iran | RCT / 54  (EG–simulation = 18; EG–serious game = 18; CG = 18) | Nursing students | Participants (nursing students) were divided into three groups to compare various methods for teaching CPR: a simulation-based CPR training group, a group that underwent CPR training incorporating a smartphone-  based serious game, and a control group that did not undergo CPR training). | Game-based mobile learning:  EG-simulation: two sessions, with each session lasting at least 45 min. EG-serious game: 2 weeks. | A 20-item, multiple-choice questionnaire for assessing CPR knowledge.  CPR skill performance was scored using a 31-item checklist. | CPR Knowledge: The simulation and serious game groups obtained significantly higher scores relative to the control group.  CPR Skill: The simulation and serious game groups obtained significantly higher scores relative to the control group. |
|  | Gamified web/  mobile learning | 5. Guti ́errez-Puertas et al. (2021)/  Spain | RCT / 184  (EG = 92;  CG = 92) | Nursing students | Participants used the Guess It (SVUAL) application to test their knowledge of advanced life support techniques and completed the Gameful Experience Scale. | Game-based mobile application / 2 hours | A test of knowledge about basic and advanced life support with 10 multiple-choice questions (four options for each question). | Knowledge: Significant differences were identified between the two groups, and a knowledge retest conducted after 3 weeks revealed significant differences. |
| Gamified web/  mobile learning | | 6. Kulakaç & Çilingir (2024)/  Turkey | RCT / 92  (EG = 45;  CG = 47) | Second-year nursing  students | Serious game-based web application for stoma care education. | Serious game-based web application / play the game for two weeks | Knowledge Test for Stoma Care and Colostomy Irrigation.  Stoma Care and Colostomy Irrigation Skill Checklists. | Knowledge: Second and retention knowledge test scores significantly higher in intervention group.  Skills: Stoma care skill scores significantly higher in intervention group. |
|  | Serious game (standalone) | 7. Blanié et al. (2020)/  France | RCT / 146  (EG = 73;  CG = 73) | Second-year nursing  students | Participants played a virtual game (LabForGames Warning) to learn about detecting clinical deterioration and improving clinical communication. | Virtual gaming simulation / > 2 hours / per session; 5 sessions | Clinical reasoning skill: script concordance tests. | For clinical reasoning skill, no significant difference was identified between the serious game–based simulation group and the traditional teaching method group, both immediately and 1 month after training. |
|  | Serious game (standalone) | 8. Chang et al. (2022)/  Taiwan | RCT / 69  (EG = 35;  CG = 34) | Fourth-year nursing students and postbaccalaureate nursing students | Participants played a board game to improve their medication knowledge. | Board game / 1 hour | Pharmacology knowledge quiz (PKQ): The 20-item PKQ covers 14 commonly prescribed medications. | The PKQ scores at post-intervention in the experimental and comparison groups were significantly higher than their pre-intervention scores. |
|  | Serious game (standalone) | 9. DemİRay & AÇIl (2024)/  Turkey | RCT / 56  (EG = 28;  CG = 28) | Second-year nursing  students | Board game for physical assessment course learning. | Board game organized / 4 weeks,  4 hours per week | Knowledge Evaluation Form for physical assessment. | Knowledge: Significant difference between pre-test and post-test knowledge scores in game group. |
|  | Serious game (standalone) | 10. Nasirzade et al. (2024)/  Iran | RCT / 42  (EG = 21;  CG = 21) | Fifth-semester nursing students | Serious game (BAM Game) for burn patient assessment. | Serious game / play the game for two weeks | Burn assessment knowledge test.  Objective Structured Clinical Examination (OSCE). | Knowledge: Intervention group knowledge scores significantly higher than control group.  Skills: Intervention group skill scores significantly higher than control group. |
|  | Serious game (standalone) | 11. Ordu & Çalışkan (2023)/  Turkey | RCT / 102  (EG = 51;  CG = 51) | First-year nursing students | Participants engaged in a virtual gaming simulation focused on nursing diagnosis, goal setting, and prioritization of diagnoses in cases of chronic obstructive pulmonary disease. | Kahoot!-based  gamification in pharmacology / 60 min | In the first section, 10 are for nursing diagnosis and 10 are for goal setting.  In the second section of the game, each student was asked to rank 10 nursing diagnoses in order of priority.  The virtual gaming simulation evaluation form consists of 15 questions. | Knowledge: The mean scores for nursing diagnosis, goal setting, and total knowledge were significantly higher in the intervention group than in the control group. |
|  | Serious game (standalone) | 12. Verkuyl et al. (2017)/  Canada | RCT / 47  (EG = 23;  CG = 24) | Fourth-year nursing  students | Participants engaged in a virtual gaming simulation to improve their pediatric nursing care knowledge, pediatric nursing care self-efficacy, and satisfaction with their learning experience. | Virtual gaming /  60 to 90 min | Pediatric Nursing Care Knowledge Test: A validated 10-item multiple-choice assessment measuring pediatric postoperative appendectomy care competencies, administered pre- and post-intervention.  Pediatric Skills Self-Efficacy Survey: A 9-item instrument utilizing a 6-point Likert scale. | Pediatric nursing care knowledge and self-efficacy: Significant gains were demonstrated only by participants in the gaming intervention group. |
|  | Gamified  simulation-based learning | 13. Aktaş et al. (2024)/  Turkey | RCT / 72  (EG = 36;  CG = 36) | First-year  nursing students | The escape room game was played once with the intervention group. | Escape room game / 6 minutes | Parenteral Drug Administration Information Test (PDAIT).  Parenteral Drug Administration Sub-Checklists (PDASC). | Knowledge: Post-test knowledge scores significantly higher in intervention group.  Skills: Intervention group showed significantly higher skill scores for drug withdrawal from ampoule, subcutaneous injection, intradermal injection, and intravenous push drug administration. |
|  | Gamified  simulation-based learning | 14. LeFlore et al. (2012)/  United States of America | RCT / 93  (EG = 46;  CG = 47) | Senior nursing students | Participants played a virtual game focused on pediatric respiratory conditions, including bronchiolitis, cystic fibrosis, pneumonia, and asthma. | Virtual gaming  simulation / 3 hours | Knowledge acquisition was assessed using a 10-item multiple-choice test.  Knowledge application was measured using objective structured clinical examinations: The infant simulator was used to score each checklist and to record the time taken for interventions. | Knowledge: A significant difference in knowledge acquisition was identified between the control and experimental groups.  Knowledge application: Significant differences in time taken to complete critical nursing tasks were identified between the study groups, with the experimental group performing these tasks more quickly in the objective structured clinical examinations. |
|  | Gamified  simulation-based learning | 15. Ma et al. (2021)/  China | RCT / 104  (EG = 51;  CG = 53) | Sophomore  nursing students | Participants in the intervention group played a disaster nursing game called “Brave the Wind and Wave” in a simulated training room. | Simulation-based learning/ 16 class hours  110 min/5 sessions  six groups  8–10 students / per group | The Questionnaire of Disaster Rescue Ability scale contains 33 items for assessing three dimensions: cognition (10 items), skills (14 items), and affective response (nine items). | The scores for the three domains of disaster nursing competence, cognition, skill, and affective response were all significantly higher in the intervention group than in the control group. |
|  | Gamified  simulation-based learning | 16. Özkan Şat et al. (2025)/  Turkey | RCT / 120 (Escape room = 40; Role-playing = 40; Control = 40) | Nursing students | Escape room game vs role-playing for violence against women assessment skills and attitudes. | Escape room game vs role-playing / 60 min | Knowledge Test on  Violence Against Women.  Scale for Attitudes Toward Violence. | Knowledge: Both escape room and role-playing groups scored significantly higher than control group at both time points.  Attitudes: At one-month follow-up, intervention groups showed more contemporary, non-approving attitudes toward violence compared to control group. |
|  | Gamified  simulation-based learning | 17. Zhao et al. (2025)/  China | RCT / 103  (EG = 51;  CG = 52) | Third-year nursing students | Theme game-based learning vs situational simulation for psychiatric violence risk assessment and response | Simulation-based learning / six sub-theme games were conducted across two class sessions | Knowledge test Objective Structured Clinical Exam (OSCE). | Knowledge: Intervention group knowledge scores significantly higher than control group.  Skills: Intervention group achieved significantly higher total OSCE scores. |
|  | Gamified flipped classroom /  animation | 18. Elzeky et al. (2022)/  Egypt | RCT / 128  (EG = 64;  CG = 64) | Nursing students | Participants were taught using a gamified flipped classroom model to enhance their fundamental skill competence and learning motivation. | Gamified flipped classroom / 6 weeks | Fundamentals of nursing knowledge tests: Three formative quizzes for each timepoint were developed by the researchers.  Fundamentals of nursing skill observation checklists.  Intensity of preparation: Three items are scored as two dichotomous items. | Skills knowledge: A significant difference was identified between the two groups in the study.  Skill performance: A significant difference was identified between the two groups in the study.  Intensity of Preparation: A significant difference was identified between the two groups in the study. |
|  | Gamified flipped classroom /  animation | 19. Inangil et al. (2022)/  Turkey | RCT / 70  (EG = 35;  CG = 35) | Nursing students | Participants received instruction incorporating animation and gamification in an online distance education course on diabetes nursing to improve their knowledge and motivation. | Animation and Gamification in Online (Kahoot) / two-course hours | Diabetes Nursing Course Knowledge Test: The test comprises 40 multiple-choice questions. | Diabetes nursing course knowledge: A significant difference was identified between the two groups in the study, with the experimental group achieving higher knowledge scores. |

*Note.* EG = experimental group; CG = control group; RCT = randomized control trial.
